# 

**Published:** 1909-12-11

**Authors:** 


### BOOKS RECEIVED.

Messrs. Methuen.

"The Mediæval Hospitals of England." By R. M. Clay.

Edward Arnold.

"A Text-book of Medical Treatment." By W. Calwell, M.A., M.D.

J. W. Arrowsmith (Bristol).

"Industrial Diseases and Accidents." By W. J. Greer, F.R.C.S., D.P.H.I.

### Gifts and Bequests.

Mr. John Perry has left by his will £50 each to the Great Northern Central Hospital, the Royal Hospital for Diseases of the Chest, the Royal London Ophthalmic Hospital, the London Throat Hospital (for Diseases of the Throat, Nose, and Ear), and to the Hospital for Women, Soho Square.

Mr. Allgood Edward Smith, who left, subject to certain legacies, his estate to his wife and brother, requested his brother to leave all the property which he should acquire under this will (and if his wife should survive his brother he requested her instead of leaving the property to his brother, to make a like disposition) to the Queen Alexandra's Davos Sanatorium and to two other charities.

The will of Dr. Thomas Joseph Cantrell, M.D., of Hyde Park Square, W., who died on July 2, aged eighty-two, has been sworn at £69,177 gross and £68,543 net. The will is at present subject to the action "Dommett and others *v.* Moser and others," now pending in the Probate Division of the High Court.

Queen Charlotte's Hospital has received a legacy of £50 under the will of the late Mrs. Greenhill. This is the only legacy received by the hospital this year. During the past three years the receipts from legacies have been abnormally low, for while the average was formerly about £2,000 per annum, the amount received in 1907 was £530 and in 1908 £311 only.

### THE QUESTION BOX

**SPECIAL NOTICE.**—Questions of interest affecting the honorary medical staff and hospital administration in all departments are invited. When any point arises we hope our readers will formulate it in a question and so co-operate to make this column of real value and interest. In this way the experience of the best-managed hospitals should be made helpful to the whole hospital world. We shall welcome the contribution of both questions and answers.

**N.B.**—The publication of an answer in this column does not necessarily preclude the publication of subsequent answers to the same question, for a question may involve points which require to be threshed out and fully discussed. Answers, if published, will be paid for at scale rates.

### HOSPITAL ATTENDANCE OF HONORARY MEDICAL STAFF.

### An Obsolete Plan.

Question 2: Is it allowable that the attendance book of the Honorary Medical Staff shall be kept by the porter at the gate who makes the entries and so saves the trouble to the Visiting Staff?

A Manchester View.

Answer: Allowable, but not likely to be satisfactory.

By Mr. W. G. Carnt, General Superintendent and Secretary, Manchester Royal Infirmary.

I do not consider an entry by the porter so satisfactory as the doctors' own signatures, and I believe many doctors who would not mind making a personal entry in an attendance book would resent an entry by a porter, but there is no reason other than this why the porter should not record the attendances, excepting that he is frequently busy answering inquiries, etc., and there is more chance of the record being inaccurate.

A Birmingham View.

Answer: No.

By Mr. Howard J. Collins, House Governor, Birmingham General Hospital.

No. It would, I think, be most objectionable to allow a porter to enter such records for the senior members of the staff.

A Leicester Practice.

Answer: Yes.

By Mr. Harry Johnson, House Governor and Secretary, Leicester Infirmary.

At this hospital such a book is and has been for some years kept by the porter on duty at the entrance hall. The hon. medical staff have always appreciated the spirit in which this book was introduced, and have assisted in a true record being kept. Similar books are also kept for the resident medical staff (who themselves enter their times of departure and return) and all the staff of the institution, including officials, porters, scrubbers, etc. These records have on occasions proved most useful.

### FIRE IN HOSPITALS.*

### Fire Drill.

Question 1: What are the principal directions for drill in a hospital of over 100 beds which is supplied with the usual appliances?

A Birmingham View.

By Mr. Howard J. Collins, House Governor, Birmingham General Hospital.

In the event of a fire in the hospital the essential regulation should be that communication by telephone or otherwise should be immediately made to the central switch-room or porters' office, and that then the resident medical and nursing staffs should confine their attention to the care of the patients who may be affected, while the porters should deal with the actual outbreak under the direction of the resident house governor, superintendent, or secretary. The weekly drill should be confined to the male servants, and should consist more particularly, beyond the understanding of the use of a fire hose, of a full knowledge of the positions of the various appliances.

A Leicester View.

By Mr. Harry Johnson, House Governor and Secretary, Leicester Infirmary.

Three principles should always be kept prominently in view:

1. Immediate centralisation of all efforts at the seat of outbreak.
2. The safety of the patients.
3. The localisation of the outbreak.

While it is obvious that any general rules in case of fire must be invaluable, it should be impressed on the staff that such rules must be varied according as local circumstances surrounding the area of outbreak may demand; stress should therefore be laid on the fact that above all else great judgment is called for in first dealing with the outbreak.

The Principal Directions.

(1) The person first to discover the outbreak to communicate immediately in person if near, or by telephone if in a distant part, to the porters' office intimating precisely the seat of the outbreak. (2) The porter receiving the intimation immediately to ring the fire bell and to communicate with the principal officials on the inter-communication system. (3) The local fire brigade to be communicated with and the nearest fire alarm broken as a precautionary measure. (4) Every male servant immediately to proceed to the seat of the outbreak. (5) Telephonic communication to be made to the adjacent wards for nurses. (6) The house governor and secretary, or, in his absence, the senior resident medical officer on duty immediately to take charge of the efforts for checking the outbreak. (7) The lady superintendent similarly to take charge and direction of efforts for the safety of patients with the assistance of the resident medical staff. (8) The male servant first arriving to proceed to connect the fire hose with the standpipe, or to use the chemical-fire apparatus, of which there is a liberal supply. (9) A porter to station himself at the nearest telephone to receive and convey messages and instructions. (10) Lift attendant to bring nearest lift to the floor on which the outbreak occurs. (11) All convalescent patients and all patients whose condition does not absolutely debar such a course immediately to obey the sisters' instructions as to quitting the ward for a place of safety.

### Removal of Patients in Case of Fire.

Question 2: In case the ordinary passages are on fire what are the best means to remove patients from the second and third floors of a hospital?

A Leicester View.

By Mr. Harry Johnson, House Governor and Secretary, Leicester Infirmary.

The reply to this question depends so much on the varying circumstances attached to each building. If the building is an old one, and there is no emergency staircase, and the approach to the ward becomes impassable

* A Special Committee of the King's Fund, after going fully into this subject, published a report thereon in July 1907. This report issummarised on another page.—Ed. The Hospital.

except at great risk, resort must be made as a last emergency to

1. The jumping sheet.
2. The canvas shoot, if such be installed, but recourse to these means would in all human probability be obviated by the arrival of the fire brigade with their escape, which, unless the institution is very far from town, would have arrived within a very few minutes of the outbreak.

General Remarks on the Leicester Infirmary.

In the modern wards adequate emergency exits are provided, and the building being of concrete and the floors of terrazzo, there is practically no imminent danger to the patients in the case of an alarm. A fire could certainly be confined to one ward, and it is difficult to conceive both general and emergency staircases being impassable. In the older wards, except on one floor, there is no third floor occupied by patients, so that the process of removal of patients in case of fire is rendered less arduous and dangerous than might be the case. The floors of the older wards are, however, of wood. The plan of construction of the wards is such that all wards immediately lead on to the main corridors 7 feet in width, the corridors being approached by a wide stone staircase (4.6 feet) at either end. Except in the case of one or two of the smaller and older wards all the wards possess a second exit in case of emergency.

Numerous fire hydrants are placed in close proximity to the respeective buildings—a 6-inch pipe from the Corporation main supplying the institution through a 4-inch pipe within the grounds. The hydrants are regularly tested.

The out-patients' department, the new nurses' home, the laundry, the boiler house and pump room, the mortuary, etc., are separate buildings, and a fire would thus be confined to the building in which it broke out. No danger to patients would result from a fire in any of these departments.

THE

### GRADUATES' MEDICAL SCHOOLS.

DIARY FOR THE WEEK, DECEMBER 13 to 18.

### ST. JOHN'S HOSPITAL FOR DISEASES OF THE SKIN, Leicester Square, W.C.

Chesterfield Lectures.—At the Hospital on Thursday evenings at 6 p.m., each lecture followed by clinical demonstrations :—

**Dec. 16, Ul-erythema:** I., Centrifugum; II., Telangiectic; III., Ophryogenes.

### THE HOSPITAL FOR SICK CHILDREN, Great Ormond Street, W.C.

**At 4 p.m.—Dec. 16,** Dr. Still, **Epilepsy.**

### MEDICAL GRADUATES' COLLEGE AND POLYCLINIC, 22 Chenies Street, W.C.

**At 4 p.m.—Dec. 13,** Dr. James Galloway, **Clinique (Skin).**

**At 5.15 p.m.—Dec. 13,** Dr. Fitzgerald Powell, **Some Diseases of the Larynx, with Special Reference to Hoarseness and Loss of Voice.**

**At 4 p.m.—Dec. 14,** Dr. W. Ewart, **Clinique (Medical).**

**At 5.15 p.m.—Dec. 14,** Dr. Percy Kidd, **Some Points in the Clinical History of Pneumonia.**

**At 4 p.m.—Dec. 15,** Mr. T. H. Openshaw, **Clinique (Surgical).**

**At 5.15 p.m.—Dec. 15,** Dr. E. I. Spriggs, **The Physiology of the Appetite: wi.h Special Reference to the Arrangement of Meals and the Use of Tonics.**

**At 4 p.m.—Dec. 16,** Sir Jonathan Hutchinson, **Clinique (Surgical).**

**At 5.15 p.m.—Dec. 16,** Dr. Robert Hutchison, **Some Conditions which Simulate Dyspepsia.**

**At 4 p.m.—Dec. 17,** Dr. St. Clair Thomson, **Clinique (Throat).**

### LONDON SCHOOL OF CLINICAL MEDICINE, Seamen's Hospital, Greenwich, S.E.

**At 2.15 p.m.—Dec. 13,** Sir Dyce Duckworth, **On the Diatheses.**

**At 2.15 p.m.—Dec. 16,** Sir Wm. Bennett.

### THE POST-GRADUATE COLLEGE, West London Hospital, Hammersmith, W.

**At 10 a.m.—Dec. 13 and 16,** Surgical Registrar, **Demonstration.**

**At 10 a.m.—Dec. 17,** Medical Registrar, **Demonstration.**

**At 12 noon.—Dec. 16,** Dr. Bernstein, **Pathological Demonstration.**

**At 12.15 p.m.—Dec. 14,** Dr. Pritchard, **Practical Medicine.**

**At 12.15 p.m.—Dec. 15** and **18,** Dr. Grainger Stewart, **Practical Medicine.**

**At 5 p.m.—Dec. 13,** Mr. Baldwin, **Practical Surgery—VI.**

**At 5 p.m.—Dec. 14,** Dr. R. Morton, **X-ray Examination of the Urinary System.**

**At 5 p.m.—Dec. 15,** Mr. Pardoe, **Bacterial Infections of the Urinary Organs.**

**At 5 p.m.—Dec. 16,** Mr. Dunn, **Purulent Conjunctivitis.**

**At 5 p.m.—Dec. 17,** Mr Etherington Smith, **Clinical Lecture.**

### NORTH-EAST LONDON POST-GRADUATE COLLEGE, Prince of Wales's General Hospital, Tottenham, N.

**At 4.30 p.m.—Dec. 14,** Dr. A. H. Pirie, **The Treatment of Ringworm by X-Rays.**

**At 4.30 p.m.—Dec. 15,** Dr. T. R. Whipham, **Idiocy and Allied Conditions in Childhood.**

### CENTRAL LONDON THROAT AND EAR HOSPITAL, Gray's Inn Road, W.C.

**At 4.30 p.m.—Dec. 16,** Dr. Andrew Wylie, **Nasal and Aural Hæmorrhage.**

### NATIONAL HOSPITAL FOR THE PARALYSED AND EPILEPTIC, Queen Sq., Bloomsbury, W.C.

**At 3.30 p.m.—Dec. 14,** Sir Wm. Gowers, F.R.S., **Clinical Lecture.**

**At 3.30 p.m.—Dec. 17,** Dr. T. Grainger Stewart, **Hysterical Paralysis.**

### ROYAL SOCIETY OF MEDICINE, 20 Hanover Square, W.

**At 5.30 p.m.—Dec. 14, Medical and Surgical Sections:** Joint debate on "The diagnosis and treatment of duodenal ulcer," opened by Mr. B. G. A. Moynihan, followed by Sir T. Lauder Brunton, Bt., Mr. Herbert Waterhouse, Mr. W. Hale White, and others. Members wishing to speak in the debate are invited to send in their names to one of the Secretaries of the Surgical Section, Mr. W. G. Spencer or Mr. J. Hutchinson.

**At 5 p.m.—Dec. 15. Special General Meeting of Fellows.**

**At 7.30 p.m. (for 8). Annual Dinner of the Society:** Applications for tickets (price 7s. 6d., not including wine) should be accompanied by cheque and sent in at once. Those who apply after Monday, December 13, will be placed at the "late tables," and their names will not be on the printed list.

**At 5 p.m.—Dec. 16. Dermatological Section:** Dr. T. D. Savill: "Sclerodermia"; Dr. George Pernet: "Sections of melanosis cutis"; Mr. J. MacDonagh: "Trichoepithelioma papulosum"; Mr. Graham Little: (1) "Telangiectatic lupus erythematosus"; (2) "Tertiary syphilis"; Mr. G. W. Dawson: (1) "Acne keloid"; (2) "Bazin's disease"; (3) "Case for diagnosis."

**At 8.30 p.m.—Dec. 17. Electro-Therapeutical Section:** Dr. N. S. Finzi: "Radium in the treatment of malgignant growths."

THE HOSPITAL December 11, 1909.

*Name* ..................................

*Address* ..................................

..................................

**This Coupon must accompany manuscript or contributions intended for The Hospital.**

